# Supplementary material for: The Impact of Frailty Components and Preoperative Mechanical Cardiac Support Changes with Time after Heart Transplantation
Source: Biomedicines. 2024 May 17;12(5):1114. doi: 10.3390/biomedicines12051114 (PMC11118029; doi:10.3390/biomedicines12051114)
Supplement: Supplementary file 1 [file biomedicines-12-01114-s001.zip › biomedicines-2985799-supplementary.pdf]

The impact of frailty components and preoperative mechanical cardiac support changes with time after heart transplantation

Rita Szentgróti, Dmitry Khochanskiy, Balázs Szécsi, Flóra Németh, András Szabó, Kinga Koritsánszky, Alexandra Vereb, Zsuzsanna Cserép, Balázs Sax, Béla Merkely, Andrea Székely

SUPPLEMENTARY MATERIAL

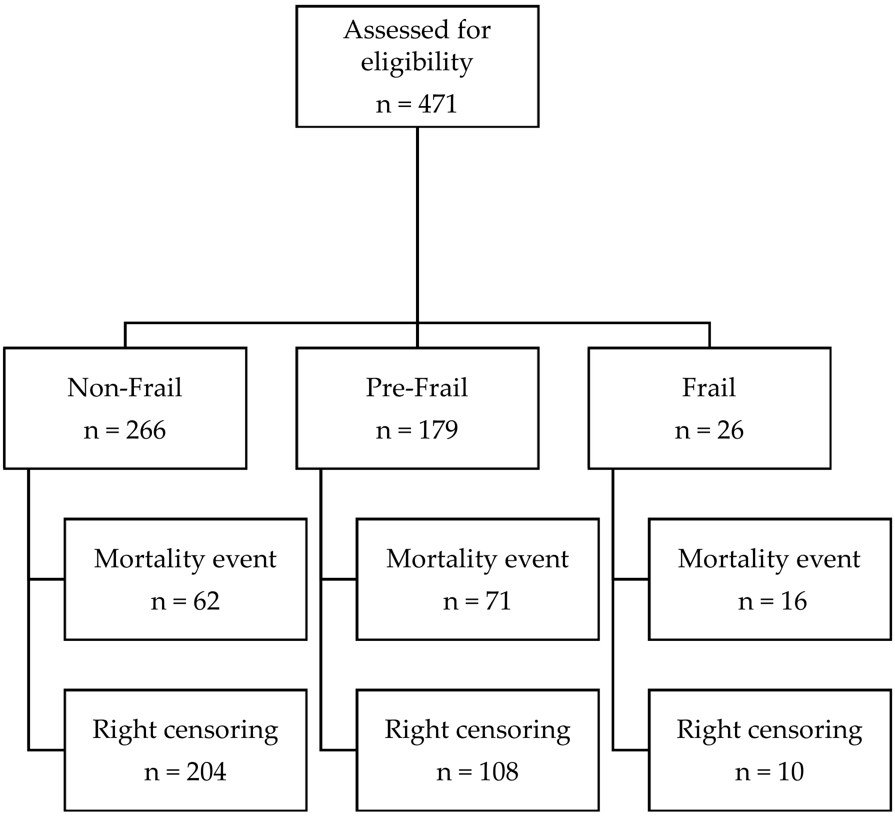

Figure S1. CONSORT statement of the study

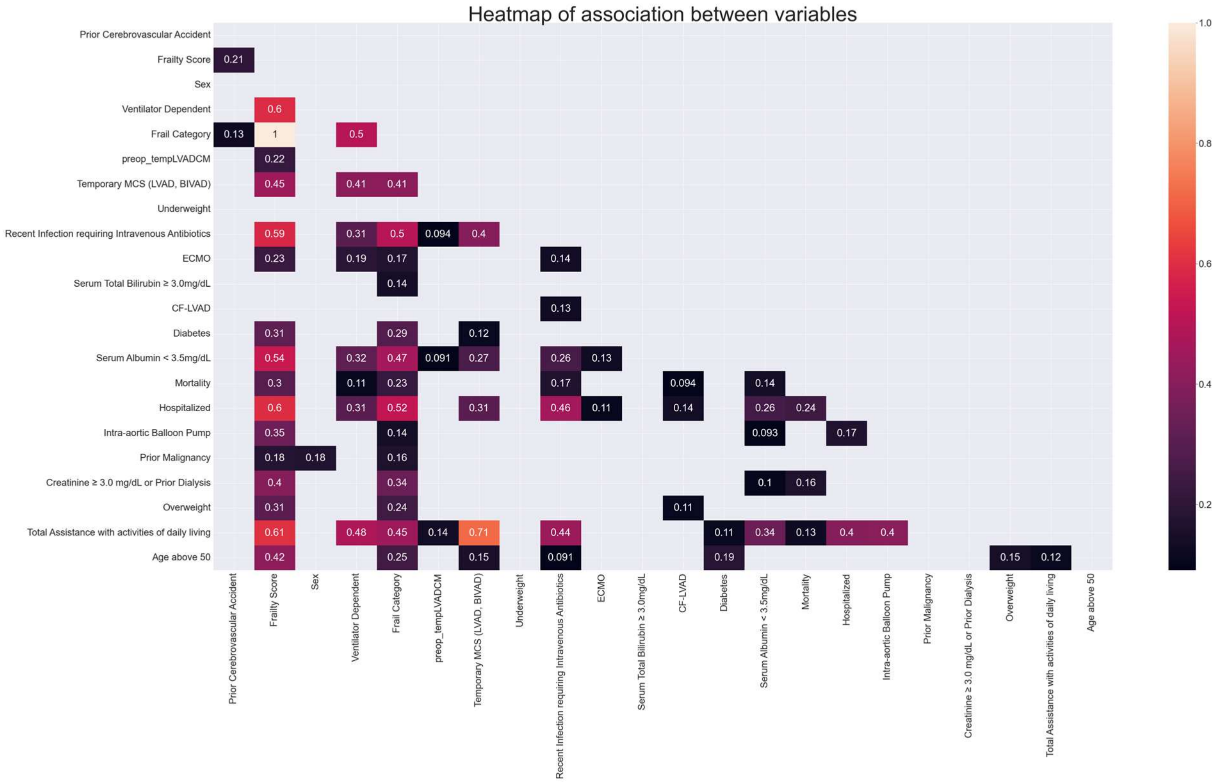

Figure S2. Cramer's V association between frailty components.
CF-LVAD: continuous flow left ventricular device, ECMO: extracorporeal membrane oxygenation

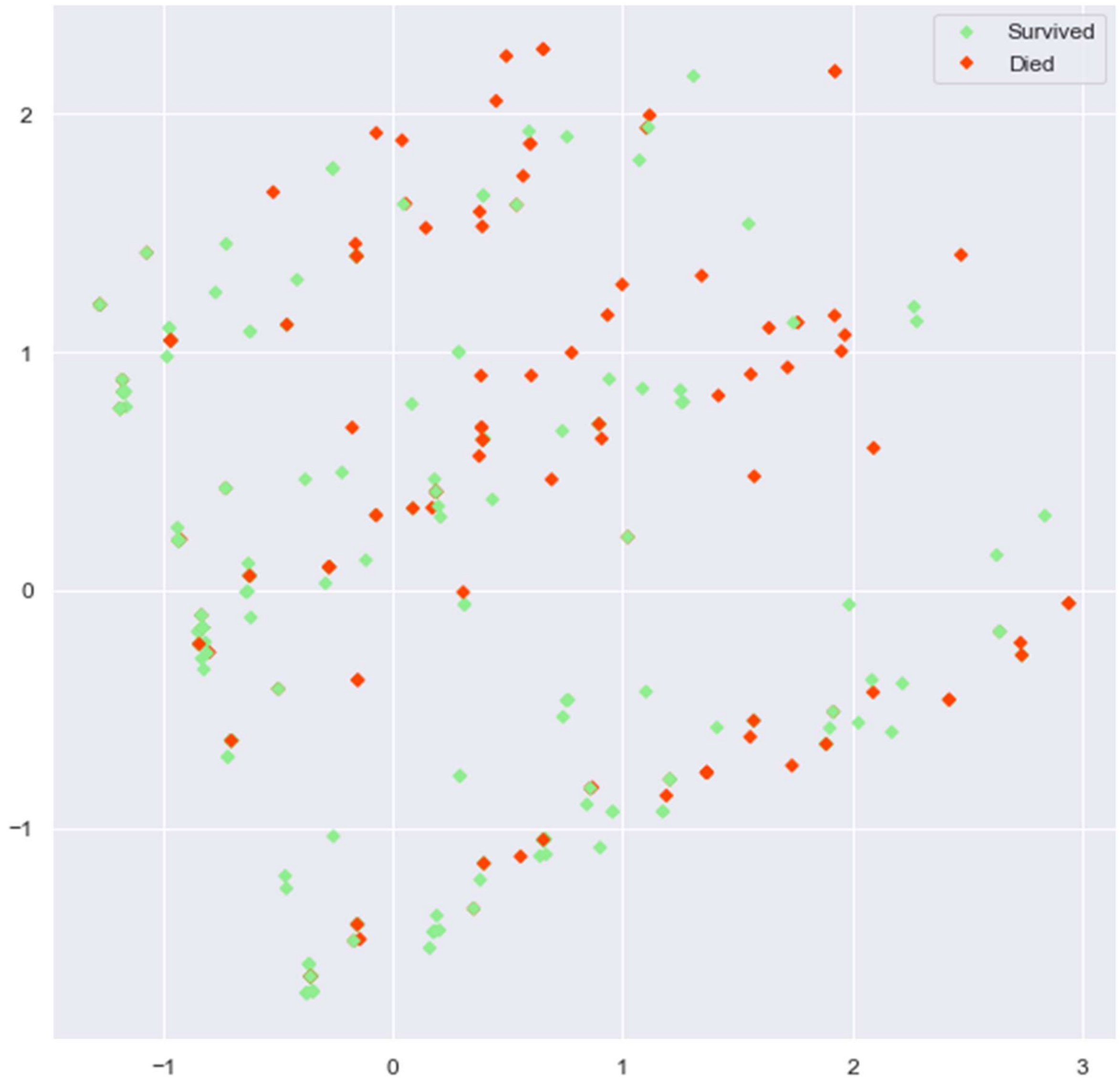

Supplement Figure S3. Principal component analysis with two principle vectors for frailty score components
Explained variation per principal component: Principle Component 1 - 0.22; Principle Component 2 - 0.15

Analyzing components of the OHT frailty screening tool with principal component analysis showed that 11 principle components were needed to capture more than 95% of the variance of the data, with a smooth plot of cumulative variance over the number of principle components. Two components, used for visualization, captured 35% of the variance. Patients did not form defined clusters, and the whole distribution was uniform, with a slight increase in the prevalence of mortality with higher values of the principal components, so components of the OHT frailty screening tool can predict outcomes with fair reliability. (Figure S3)

Table S1. Mortality at different time points for statistically significant components of the frailty score, defined in multivariable Cox model for different time periods

**Variables**		**Mortality**				
		**30 days**	**1 year**	**2 years**	**5 years**	**Total**
**Age above 50**	**Mortality event**	38	65	75	82	95
	**Censoring**	0	0	8	66	187
	**p**	0.0315	0.0193	0.0072	0.0627	0.0353
**Creatinine ≥ 3.0 mg/dL or Prior Dialysis**	**Mortality event**	18	38	46	50	53
	**Censoring**	0	0	2	29	64
	**p**	0.4006	<0.005	<0.005	<0.005	<0.005
**Hospitalized**	**Mortality event**	32	57	65	75	84
	**Censoring**	0	0	2	29	99
	**p**	0.0412	<0.005	<0.005	<0.005	<0.005

n – cumulative mortality, **%** - percentage of patients who died from the initial cohort size, **p** – statistical significance from multivariate time-dependent Cox hazard model

Table S2. Follow-up hazard ratios of the multivariable Cox- model for frailty score components at different time points

Variables		Time periods				
		30 days	1 year	2 years	5 years	Total
Variables significant at least at one time point						
c-index		0.71	0.69	0.69	0.68	0.67
Age above 50	HR	1.99	1.73	1.8	1.44	1.47
	CI	1.06-3.74	1.09-2.73	1.17-2.77	0.98-2.1	1.03-2.09
	P	0.0315	0.0193	0.0072	0.0627	0.0353
Creatinine ≥ 3.0 mg/dL or prior dialysis	HR	1.29	1.87	2.18	1.97	1.78
	CI	0.71-2.35	1.22-2.86	1.47-3.23	1.36-2.84	1.26-2.52
	p	0.4006	<0.005	<0.005	<0.005	<0.005
Hospitalized	HR	1.99	1.98	1.98	2.02	1.93
	CI	1.03-3.86	1.22-3.22	1.26-3.12	1.34-3.05	1.32-2.83
	p	0.0412	0.0058	<0.005	<0.005	<0.005
Statistically insignificant variables						
Overweight	HR	1.86	1.34	1.18	0.99	1.02
	CI	0.96-3.61	0.8-2.25	0.72-1.94	0.61-1.6	0.65-1.61
	p	0.0652	0.262	0.5032	0.9515	0.9166
Underweight	HR	2.05	1.24	1.07	1.16	1
	CI	0.62-6.84	0.38-4.01	0.33-3.47	0.42-3.22	0.37-2.76
	p	0.2422	0.7241	0.9051	0.7695	0.9945
Serum albumin < 3.5 mg/dL	HR	1.32	1.27	1.19	1.2	1.21
	CI	0.68-2.54	0.78-2.08	0.75-1.88	0.78-1.84	0.82-1.81
	p	0.4119	0.3323	0.4717	0.4015	0.3394
Serum total bilirubin ≥ 3.0 mg/dL	HR	1.32	0.98	0.83	0.67	0.7
	CI	0.31-5.52	0.31-3.15	0.26-2.66	0.21-2.11	0.26-1.91
	p	0.7069	0.9788	0.7569	0.4904	0.4865
Diabetes	HR	0.93	1.2	1.15	1.18	1.21
	CI	0.51-1.72	0.76-1.88	0.75-1.76	0.8-1.75	0.85-1.74
	p	0.8261	0.4359	0.5181	0.4087	0.2939
Ventilator dependent	HR	1.29	1.33	1.46	1.23	1.29
	CI	0.43-3.82	0.6-2.94	0.69-3.09	0.6-2.51	0.65-2.55
	p	0.6495	0.4878	0.3213	0.5745	0.4615

**Prior cerebrovascular accident**	**HR**	1.64	1.21	1.23	1.2	1.24
	**CI**	0.79-3.42	0.65-2.23	0.7-2.18	0.72-2.02	0.77-2
	**p**	0.1844	0.5466	0.4703	0.4841	0.369
**Prior malignancy**	**HR**	0.32	0.77	0.66	0.66	0.64
	**CI**	0.04-2.32	0.31-1.91	0.27-1.62	0.29-1.51	0.3-1.38
	**p**	0.2572	0.5748	0.3623	0.3253	0.2525
**Total assistance with activities of daily living**	**HR**	0.87	1.2	1.3	1.25	1.17
	**CI**	0.33-2.28	0.6-2.42	0.67-2.52	0.68-2.3	0.65-2.09
	**p**	0.7767	0.6014	0.4381	0.4688	0.6071
**Recent infection requiring intravenous antibiotics**	**HR**	1.84	1.4	1.28	1.25	1.31
	**CI**	0.93-3.65	0.83-2.39	0.77-2.12	0.78-1.99	0.85-2.03
	**p**	0.0797	0.2105	0.3411	0.3474	0.226
